# Some Spinal Cord Lesions.—VI

**Published:** 1911-09-09

**Authors:** 


### Neurology.

### SOME SPINAL CORD LESIONS.—VI.

### COMPRESSION PARAPLEGIA.

Compression of the spinal cord in the cervical or dorsal regions gives rise to loss of power in the legs, spastic rigidity, and exaggerated reflexes. At the seat of compression the cord is softened, myelitis set up, and degeneration follows in the sensory columns above and in the pyramidal tracts.

The chief causes of compression are: caries of the vertebræ, tumours in the bones, tumours of the meninges, pachymeningitis, meningeal hæmorrhage, aneurysm of the aorta.

The symptoms vary according to the site, the extent, and the cause of the compression. The nerve roots may be compressed as well as the cord.

The onset is gradual, except in the case of hæmorrhage, when it is very sudden. The earliest symptoms are pain in the spine and along the nerves, whose roots are compressed, followed by loss of power and spasticity of the legs. In a small proportion of spinal caries the predisposing cause may be a sudden jump or other movement causing fracture of the diseased bones in a person hitherto regarded as sound, and this leads to an absolutely sudden paraplegia.

In a well-marked case of compression of the mid-dorsal region of the spinal cord the chief symptoms are: loss of power in the legs, rigidity and spasmodic contractions of the muscles of the leg; the arms are not affected except in very rare cases in which the caries involves the lower cervical vertebræ.

There is pain of a burning or neuralgic character in the area of the distribution of the nerves whose nerve roots are directly involved by the caries. A varying degree of anæsthesia exists below the seat of the lesion; in some cases there is very little or no anæsthesia, but in others there is absolute analgesia and anæsthesia of the legs and lower part of the trunk.

The knee-jerks are increased, ankle and patellar clonus are present, and the plantar reflexes are extensor in character, provided the lumbar enlargement remains intact. It should be remembered that if there is complete compression of the lumbar cord the reflexes will be absent.

Bed sores are common. No atrophy of the muscles of the legs occurs except from disuse, when the lumbar enlargement is not affected; but if the tuberculous masses compress the lumbar part of the cord there will be atrophy of the leg muscles simulating peripheral neuritis.

There will be no reaction of degeneration when there is no wasting, but marked R.D. when the lumbar enlargement is destroyed.

There may be involuntary passage of urine and fæces; this impairment of function, as a rule, affects the bladder before the rectum, generally causing retention with overflow. The special senses are not affected.

If the cervical region is compressed, in addition to the above-mentioned symptoms, the arms are paralysed, there is atrophy of the muscles of the hands and arms, and these muscles give the reaction of degeneration. In all cases of paraplegia, associated with pain along the course of the nerve roots and anæsthesia below the seat of the lesion, the vertebræ must be carefully examined for signs of disease.

In spinal caries there may be deformity of the spine, pain on movement, and deep local tenderness, though external deformity is not invariably found; if in the cervical region thickening of the tissues around the spine may be detected. Signs of tuberculous disease in other parts of the body would also be in favour of spinal caries. It is the commonest cause of paraplegia in children.

In malignant disease nodules of growth may be detected attached to the vertebræ or be found in other parts of the body. A man suffering from paraplegia which was considered to be due to new growth developed a nodule in the frontal bone which rapidly increased in size and helped to confirm the diagnosis. The pain is more severe than it is in caries, and the deformity is less. The primary growth may already be known to exist in the stomach, breast, and so on.

Aneurysm of the aorta is a very rare cause of paraplegia from compression of the spinal cord. Pain in the back is very severe at first from erosion of the bones, and later from compression of the nerve roots. If, in addition to signs of compression, there is a local pulsating tumour of the back in a patient who has suffered from syphilis and has been engaged in a laborious occupation, the diagnosis of compression by an aneurysm should not present much difficulty. It may be confirmed by the *x*-rays.

